# Soil-borne fungi influence seed germination and mortality, with implications for coexistence of desert winter annual plants

**DOI:** 10.1371/journal.pone.0224417

**Published:** 2019-10-31

**Authors:** Yue M. Li, Justin P. Shaffer, Brenna Hall, Hongseok Ko

**Affiliations:** 1 School of Natural Resources and the Environment, University of Arizona, Tucson, Arizona, United States of America; 2 Arizona-Sonora Desert Museum, Tucson, Arizona, United States of America; 3 School of Plant Sciences, University of Arizona, Tucson, Arizona, United States of America; 4 College of Public Health, University of Arizona, Tucson, Arizona, United States of America; 5 Department of Ecology and Evolutionary Biology, University of Arizona, Tucson, United States of America; Brigham Young University, UNITED STATES

## Abstract

Soil-borne fungi influence coexistence of plant species in mesic environments, but much less is known about their effects on demographic processes relevant to coexistence in arid and semi-arid systems. We isolated 43 fungal strains that naturally colonize seeds of an invasive winter annual (*Brassica tournefortii*) in the Sonoran Desert, and evaluated the impact of 18 of them on seed germination and mortality of *B*. *tournefortii* and a co-occurring native annual (*Plantago ovata*) under simulated summer and winter temperatures. Fungi isolated from *B*. *tournefortii* seeds impacted germination and mortality of seeds of both plant species *in vitro*. Seed responses reflected host-specific effects by fungi, the degree of which differed significantly between the strains, and depended on the temperature. In the winter temperature, ten fungal strains increased or reduced seed germination, but substantial seed mortality due to fungi was not observed. Two strains increased germination of *P*. *ovata* more strongly than *B*. *tournefortii*. In the summer temperature, fungi induced both substantial seed germination and mortality, with ten strains demonstrating host-specificity. Under natural conditions, host-specific effects of fungi on seed germination may further differentiate plant species niche in germination response, with a potential of promoting coexistence. Both host-specific and non-host-specific effects of fungi on seed loss may induce polarizing effects on plant coexistence depending on the ecological context. The coexistence theory provides a clear framework to interpret these polarizing effects. Moreover, fungi pathogenic to both plant species could induce host-specific germination, which challenges the theoretical assumption of density-independent germination response. These implications from an *in vitro* study underscore the need to weave theoretical modeling, reductive empirical experiments, and natural observations to illuminate effects of soil-borne fungi on coexistence of annual plant species in variable desert environments.

## Introduction

One major endeavor in ecology is to uncover ecological factors that promote or undermine coexistence of competing species. Species coexistence is possible when stabilizing mechanisms, arising from niche differentiation, overcome the average-fitness differences between species (i.e., fitness differences averaged over broad temporal and spatial scales; [1]). Soil-borne fungi can either increase or reduce the strength of stabilizing mechanisms and average-fitness differences between plant species, hence capable of substantially influencing coexistence of plant species [2,3].

Stabilizing mechanisms occur when interspecific differences at the population level results in niche differentiation with respect to resource acquisition, tolerance to natural enemies, or response to variation in a physical environment [4–8]. The outcome is an intensification of intraspecific relative to interspecific density dependent feedback, promoting coexistence [1]. Soil-borne fungi may impart host-specific effects that limit population growth when a given species becomes relatively common, and favor population growth when that species becomes relatively rare, thus enhancing stabilizing mechanisms [9–18]. For instance, host-specific effects of fungi on seedling recruitment promote plant diversity in a tropical forest by primarily strengthening natural enemy partitioning, a major stabilizing mechanism [9]. Soil-borne fungi also can weaken stabilizing mechanisms: for example, pathogens carried by dominant species may limit the population recovery of species that have become relatively rare (i.e., pathogen spillover; [19]). As natural enemies, fungal pathogens can generate density dependent feedbacks (i.e., apparent competition) that interfere with the stabilizing effects that arise from resource competition (e.g., resource partitioning and the storage effect due to resource competition) [1,20,21]. In this scenario, the presence of fungal pathogens can strengthen or weaken the coexistence potential among plants, an effect that cannot be detected when effects of resource competition are examined alone (e.g., [21]).

Soil-borne fungi also can modify the average-fitness differences between plant species. When the overall impact of both general and host-specific fungal pathogens is evaluated over large-scale and long-term ecological conditions, a plant species can be either more or less resistant than other species to fungal pathogens [3]. This difference in resistance modifies the average fitness differences. A reduction in average-fitness difference makes coexistence more likely whereas an increase undermines coexistence [1]. For instance, it has been hypothesized that plant invasion may be facilitated by pathogen escape [22], through which the invasive plant species gain an average-fitness advantage over the native species because the invasive species are, on average, less attacked by pathogens. This increase in average-fitness advantage of the invasive species reduces the potential for invasive-native coexistence. Nevertheless, evidence supporting this hypothesis is limited ([3],see also [23]).

Effects of soil-borne fungi on plant demography in natural systems are studied most often in mesic environments (e.g., [17]). Much less is known about the ways in which fungi impact plant demography in natural systems of arid and semi-arid environments (but see [10,11,24]). Studies from a coastal grassland in central California [24] and the Great Basin Desert in Utah [10,25] have shown that pathogenic fungi in xeric environments can affect density dependent feedbacks among plant species and have host-specific effects under certain conditions. Theoretical work based on field evidence has suggested that a fungal pathogen of seeds can weaken coexistence between native perennial grasses and the invasive annual cheatgrass (*Bromus tectorum*) in the Great Basin region [19]. This negative effect on coexistence occurs because cheatgrass is, on average, more resistant to the pathogen than are the native grasses [19].

In warm deserts, such as the Sonoran and Chihuahuan Deserts of southwestern North America, winter annual plants form diverse communities that typically represent a large component of the standing biomass in cool-season months between November and March [26]. Winter annual plants comprise approximately half of the floristic richness in local plant assemblages in the Sonoran Desert [27], and long have been models for studying species coexistence [28–30]. However, the positive and negative impacts of soil-borne fungi on coexistence of these plants, to our knowledge, have not been studied.

Desert winter annuals normally form persistent seed banks and are sensitive to seasonal cues [31]. In particular, seeds of desert winter annuals typically experience an annual cycle with transitions between a dormant and non-dormant state: seeds become dormant or conditionally dormant in the spring, gradually lose dormancy under high summer temperatures, and become non-dormant in the autumn [32]. Seeds that survive the summer and autumn can then germinate in response to winter storms in order to grow and reproduce. Germination is sensitive to cool-season temperatures, soil moisture levels, light availability, and other abiotic factors [31,33,34]. Consequently, seed germination and mortality are key demographic factors that influence coexistence of desert winter annual species [28,31]. The Sonoran Desert differs from Mediterranean-climate grasslands and North American cold deserts by receiving substantial summer rainfall. Because fungi require moist conditions for spore germination and successful infection on hosts, the presence of both summer and winter rainfall sets the potential for fungi to be active in both seasons to influence seed mortality and germination under two different temperature regimes. This extended period of fungal activity further underscores the need for investigating the influence of fungi on species coexistence in these unique desert plant communities.

In many parts of the Sonoran Desert, winter annuals are threatened by climate shifts and invasive species [35–39]. One of these invasive species is Sahara mustard (*Brassica tournefortii*, Brassicaceae). *Brassica tournefortii* is widespread in southwestern North America and is locally common in areas of the Sonoran Desert [40]. In southwestern Arizona and southeastern California, it has become a serious threat to diverse communities of native winter annuals [35,39]. Here we isolated representative fungi from the soil seed bank of *B*. *tournefortii* in the Sonoran Desert. We then measured the effects of these fungi on fractions of seed germination and mortality, focusing on *B*. *tournefortii* and a co-occurring common native species (*Plantago ovata*, Plantaginaceae) under controlled temperature regimes consistent with summer and winter seasons.

We chose to study the effects of fungi on seed germination and mortality because fungi that infect seeds of this invasive species or co-occurring native winter annuals could influence plant interactions through at least two processes: first, by impacting the loss of seeds from the seed bank due to a) seed mortality in any season or b) germination in the wrong season (e.g., summer, when abiotic conditions limit seedling survival); and second, by increasing or reducing seed germination during the winter season, when abiotic conditions would favor seedling establishment.

Seed loss due to fungal pathogens can either promote or undermine coexistence depending on the relative dominance (i.e., average-fitness differences) of the species that are more severely attacked by the fungal pathogens [3]. *Brassica tournefortii* experienced higher seed mortality than other co-occurring annuals in southwestern Arizona, including *P*. *ovata*, over three years of naturally variable environments [33], raising the speculation that fungal pathogens may undermine its seed banks (see [41,42]). This potential influence of fungal pathogens may reduce average-fitness advantage of *B*. *tournefortii* over native winter annual species or reduce the niche overlap between *B*. *tournefortii* and the native species in ways in which fungal pathogens attack them. Both effects would lead to the promotion of coexistence between *B*. *tournefortii* and the natives.

Moreover, plant species-specific germination represents a major pathway for niche differentiation among desert winter annuals [7,28,31], which may be enhanced or weakened by certain fungi (e.g., [41]). A three-year field study in southwestern Arizona showed that *P*. *ovata* seeds had lower fractions of germination than those of *B*. *tournefortii* on a sand flat, but higher fractions on a dune, and these differences were more pronounced when the amount of first winter rainfall increased [33]. It is unclear how soil-borne fungi active in the winter rainy season may differentiate germination responses between *B*. *tournefortii* and other annual species such as *P*. *ovata*.

The differences in seed loss and seed germination in natural conditions between *B*. *tournefortii* and *P*. *ovata* led us to choose these two species as our focus, and to determine whether fungi in an *in vitro* condition might influence seed mortality and germination of warm-desert winter annual plants in a way indicative of their effects on plant coexistence. By doing so, we took the opportunity of biological invasion to assess the role of fungi in influencing plant coexistence under the theoretical framework of species coexistence [43,44]. We used a reductionist approach to assess each fungal strain *in vitro*. We then linked the findings with species coexistence theory and discussed the implications for plant coexistence by the effects of fungi uncovered in this study. More specifically, we asked the following questions through our *in vitro* experiment.

First, we evaluated whether and which fungi isolated from the invasive *B*. *tournefortii* could induce host-specific germination responses of the two plant species in a simulated winter condition. Host-specific germination responses may differentiate the response niche of the two plant species. If followed by resource or apparent competition, this differentiation in response niche may promote plant coexistence [30].

Second, we asked whether and which of these fungi could induce host-specific seed loss of *B*. *tournefortii* and *P*. *ovata* in a simulated summer condition. Host-specific seed loss of *B*. *tournefortii* may reduce its average-fitness advantage over *P*. *ovata*, facilitating native-invasive coexistence; whereas host-specific seed loss of *P*. *ovata* may increase the average-fitness advantage of *B*. *tournefortii*, undermining plant coexistence. Further, host-specific seed loss of both species may reduce the two species’ niche overlap in which fungi attack their seeds, promoting their coexistence. Finally, non-host-specific seed loss of both species may either promote or undermine their coexistence depending on the ecological context, which we describe in detail in the discussion.

## Methods

We collected soil that contained seeds of *B*. *tournefortii* in two sites in which that species was common in Tucson, Arizona, USA in February 2016 (TQ01: 32.25111° N, 110.75712° W, 797 meters above sea level (m.a.s.l.); TQ02: 32.23536° N, 110.75682° W, 804 m.a.s.l.). Tucson is located in the Arizona Upland subdivision of the Sonoran Desert. The area receives an average of 300 mm rainfall annually, which arrives bimodally as summer monsoons from July to mid-September and as winter storms from November to March. The average high temperature in July is 37.6°C, and the average low temperature in January is 4.3°C (https://www.wrh.noaa.gov/twc/climate/tus.php). *Brassica tournefortii* was recorded in 2003 within 5 km of the soil collection sites [45], but the species has been present in the Tucson area since at least 1968 (when the first herbarium specimen of *B*. *tournefortii* in the larger Tucson area was collected: R. Dick 164662, University of Arizona Herbarium). When our collections were made, plants at the sites appeared healthy and formed a monodominant stand in each site. The soil was dry, sandy and loamy, and representative of local soils in the area. Collections were made in public right-of-way along roads maintained by Pima County. No permit is required for collection in either site and no endangered or protected species is known to these sites.

In each site, we collected soil within 6 m of paved roads, where *B*. *tournefortii* occurred frequently. No fully developed seedpods of *B*. *tournefortii* were visible when soil samples were collected, such that seeds in these soil samples were interpreted as representing seed production prior to this recruitment season, and were either dormant or degraded at collection. A hand trowel was used to collect soil from the surface to 5 cm in depth. In each site, soil samples were collected from ten locations along a linear transect of approximately 20 m. The samples were then mixed and stored in multiple, sealed Ziploc® bags at room temperature until seeds were retrieved by flotation in April-August 2016. Seeds of *B*. *tournefortii* were identified with the aid of a stereomicroscope and retrieved with forceps. Seeds were stored in clean centrifuge tubes at laboratory conditions (25°C, low humidity) until they were processed to isolate fungi. The mixing of soil samples, and the pooling and storage of seeds could alter the composition of fungi within *B*. *tournefortii* seeds. As our aim was to identify fungal strains and evaluate their effects on seed demography individually, rather than to reveal detailed spatial variation in fungal composition and their combined impact on seeds, this potential change of composition of fungi would not affect the results presented in this study.

### Isolating fungi

We processed seeds of *B*. *tournefortii* to isolate representative fungi for seed inoculation assays. Seeds from site TQ01 were processed in November 2016. Eighty one seeds were vortexed for 3 min in 50 mL of sterile water to decrease the incidence of surface contaminants (1-min intervals; water was decanted and replaced between each interval). We used sterile water over ethanol or other sterilants to avoid damaging the seed interior, which is protected by a thin seed coat. Seeds were cut open with a scalpel under sterile conditions to score seed viability. Seeds with a white fleshy interior were considered viable. Those with a discolored (dark or yellow) and structurally degraded interior were considered degraded. We chose visual examination over a tetrazolium test to determine seed mortality because desert winter annual seeds in deep dormancy may not be adequately stained by tetrazolium [46].

Nine of the 81 seeds were degraded. Viable and degraded seeds were pooled separately. Seven pools of viable seeds (each containing 10–12 seeds) and two pools of degraded seeds (each containing 4–5 seeds) were macerated individually in a sterile 1.5-mL microcentrifuge tube with a sterile pestle. One half of each pool was suspended in 200 μl of 1X phosphate-buffered saline (PBS). The other half was suspended in 200 μl of sterile water. From each half of each pool, we isolated cultivable fungi on 2% malt extract agar (MEA) amended with ampicillin (100 μg/mL) to limit bacterial growth. We used a dilution plating approach in 12-well plates (undiluted to 10^−7^ of the original concentration in 10-fold dilutions). Plates were checked for fungal growth daily for seven days starting 24 hours after inoculation, and then less frequently until no new fungal colonies were observed. Visually unique colonies were isolated under sterile conditions onto 2% MEA amended with ampicillin in 35-mm Petri plates. We visually inspected all isolated colonies and grouped them into strains according to their color, texture and growth rate. Bacteria were observed infrequently.

Seeds from site TQ02 were processed in March 2017 as above, except for three factors. First, fungi were isolated from 40 processed seeds that were divided into four pools of viable and two pools of degraded seeds. Second, we used only PBS for suspension after seeds from site TQ01 yielded more fungal growth from PBS than water suspensions. Third, we did not amend the growth medium with antibiotics. As our goal was not a comprehensive survey of fungi, the use of MEA and the difference in processing samples from the two sites should not compromise the study as a whole. Similar approaches for isolating seed-associated fungi are described in previous work [17,47,48].

### Classification of fungi

Fungi obtained from seeds of *B*. *tournefortii* were archived as living vouchers in the Robert L. Gilbertson Mycological Herbarium at the University of Arizona (accession numbers available upon request). We extracted DNA from fresh mycelium of each fungal strain using the REDExtract-N-Amp Plant Kit (Sigma-Aldrich, St. Louis, MO, USA) following Shaffer et al. [49]. We used the polymerase chain reaction to amplify the nuclear ribosomal internal transcribed spacers and the 5.8*S* gene (ITS rDNA) and the first 600 base pairs (bp) of the large subunit (partial LSU rDNA) as a single fragment (forward primer ITS1F and reverse primer LR3) following Shaffer et al. [49]. We included water instead of template for negative controls, which were always blank. Positive PCR products were cleaned with ExoSAP-IT (Affymetrix, Santa Clara, CA, USA) following the manufacturer’s instructions, and diluted 1:2 with molecular grade water prior to sequencing. Diluted products were sequenced bidirectionally at the University of Arizona Genetics Core following Shaffer et al. [49].

We verified base calls by inspecting chromatograms in Sequencher v.5.1 (Gene Codes Corp., Ann Arbor, MI, USA). We submitted sequences to the Tree-based Alignment Selector toolkit (T-BAS, http://tbas.hpc.ncsu.edu; [50]) for phylogenetic placement and clustering based on 95, 99, and 100% sequence similarity (see [51,52]). Operational taxonomic units (OTUs) were assigned based on 95% sequence similarity. Genotypes were assigned based on 100% sequence similarity and are referred to as strains hereafter. All sequence data were accessioned at GenBank (accessions MG924996-MG925038, S1 Table). In total, the collection included 43 morphologically distinct fungal strains that represent seven orders of Pezizomycotina and belong to 14 OTUs (S1 Table). Eighteen strains were used in the inoculation experiment, which belonged to nine OTUs in five orders (Table 1). These strains represented the morphological diversity of fungi observed and included isolates from both viable and degraded seeds of *B*. *tournefortii* (Table 1).

**Table 1:** Fungal strains from seeds of *B*. *tournefortii* used in seed inoculations. Strain numbers indicate OTU designations according to 95% ITS rDNA sequence similarity, and letters indicate distinct genotypes based on 100% sequence similarity. Seeds of *B*. *tournefortii* from which the fungal strains were originally isolated are listed as viable (V), degraded (D), or both (V&D). Taxonomic assignments to genus and order levels were provided by T-BAS [50].

Strain	Source	Taxon assignment by T-BAS	Order
01A	D	*Fusarium* sp.	Hypocreales
01B	D	*Fusarium* sp.	Hypocreales
01C	D	*Fusarium* sp.	Hypocreales
01D	D	*Fusarium* sp.	Hypocreales
02A	V	*Alternaria* sp.	Pleosporales
02B	D	*Alternaria* sp.	Pleosporales
02C	V	*Alternaria* sp.	Pleosporales
02D	V	*Alternaria* sp.	Pleosporales
03A	D	*Ascochyta* sp.	Pleosporales
03B	V&D	*Ascochyta* sp.	Pleosporales
03C	V	*Ascochyta* sp.	Pleosporales
04A	D	*Fusarium* sp.	Hypocreales
04B	D	*Fusarium* sp.	Hypocreales
05	V	*Melanopsamma* sp.	Hypocreales
06	V	*Aureobasidium* sp.	Dothideales
07	V	*Fusarium* sp.	Hypocreales
08	V	*Talaromyces* sp.	Eurotiales
09	V&D	*Chaetomium* sp.	Sordariales

### Collection of seeds for inoculation trials

For seed inoculation trials we collected mature seeds of *B*. *tournefortii* and a co-occurring, native species (*Plantago ovata*) from living plants in southwestern Arizona (ca. 32.69° N, 113.83° W, 110 m.a.s.l) in March 2013. The location receives minimal anthropogenic disturbance and is adjacent to field sites where demography of *B*. *tournefortii*, *P*. *ovata* and other winter annual species was studied under natural conditions [33]. At collection, the plants were near the end of senescence with most seeds fully developed. Studies using the same approach of seed collection showed minimal seed inviability [31,33,34].

To maintain typical temperature-driven annual cycles in seed dormancy, seeds were stored outside each summer between 2013 and 2015, with exposure to ambient temperature but protection from high humidity. Otherwise, they were stored indoors at *ca*. 25°C with low humidity. Previous work showed that *B*. *tournefortii* seeds stored dry at room temperature can maintain >99% viability after ca. 3 years [53]. *Plantago ovata* seeds have higher viability rates than *B*. *tournefortii* seeds in the field [33] and high germination rates after storage for >2 years have been reported previously [54]. Therefore, our storage of seeds of both species should not noticeably affect their viability.

### Seed inoculations

In April 2017, we inoculated seeds of *B*. *tournefortii* and *P*. *ovata* with actively growing mycelium of each of the 18 focal fungal strains (Table 1). We first washed seeds as described above. We then placed seeds of both plant species, with 10 seeds of each species, in one Petri plate containing a fresh (5–10 days old) culture of a single fungal strain grown on 2% MEA. Two temperature regimes were set and controlled, each in a separate growth chamber, to simulate a summer condition (i.e., temperature fluctuating linearly between a daily high of 36°C and a daily low of 24°C) and a winter condition (i.e., a daily high and low of 20°C and 9.5°C, respectively). A control group with seeds exposed only to 2% MEA was included in each temperature regime. Five replicate plates were prepared for each fungal strain (or control) × temperature regime combination. Seeds were incubated in growth chambers in complete darkness for 13–14 days and 14–16 days in the summer and winter temperature regime, respectively (temperature readings of the two chambers are available at https://osf.io/chabj/). We then scored seed mortality and germination as described below. Previous experiments suggest that no additional germination would occur after these time periods [33]. Most fungal strains grew over the entire Petri plate and covered all seeds by the end of the experiment, suggesting sufficient duration for fungal hyphae to contact seeds in both temperature regimes.

After incubation all seeds were examined under a stereomicroscope. Those with protruding radicles were considered germinated. Non-germinated seeds were cut open. Those with softened, yellowing tissues were considered degraded and inviable (dead). The rest were considered dormant (viable).

### Statistical analyses

Throughout the statistical analyses, we emphasized presenting and interpreting our results with effect sizes and 95% confidence intervals (C.I.s) obtained from non-parametric bootstrapping. We prioritized this approach over *P*-value based significance tests for two reasons. First, an interpretation based on significance tests may veil the magnitude and thus biological importance of the results, while posing an arbitrary threshold of the significance level [55–57]. Our choice of a 95% C.I. did rely on an arbitrary threshold of the confidence level (i.e., *α* = 0.05). Nevertheless, the effect sizes and the C.I.s gave a transparent presentation of the biological importance and precision of our results. Second, our statistical analyses were designed around the following four constraints: 1. seed responses to fungi were first corrected by a response of controls (i.e., response of seeds not inoculated by any fungi); 2. the data were binomially distributed; 3. each Petri plate was considered as a random effect as seeds of both plant species were placed within; and 4. we aimed to present our results as clear, biologically interpretable effect sizes, preferably without data transformation. Significance tests within the framework of general and generalized linear mixed-effect models can address some but not all of these constraints. In contrast, a bootstrapping approach is capable of performing analyses under these constraints [58,59].

Having evaluated effect sizes and C.I.s of all variables of our interest, we reinforced our statistical inferences with significance tests. We used pairwise *t*-tests to determine whether seed responses (germination, mortality, overall loss) to each fungal strain was significantly different from the controls; and used mixed-model analysis of variance (ANOVA) to test for significant fungal strain × plant species interactions on fractions of seed germination, mortality, and overall loss. Any significant interactions would indicate that fungi could induce host-specific effects on these seed demographic responses, and that these effects depended on the identity of the fungal strain. All variables of seed responses were logit-transformed when being evaluated in these tests, which are based on general linear models.

When estimating confidence intervals and evaluating *P*-values of significance tests, we chose not to control for familywise error rate due to the controversy around the necessity of this control [60–62]. Specifically, we do not agree with the assumption that all null hypotheses in our experiment (zero effect on germination/mortality by any fungal strain) would simultaneously be true, especially given the results of our mixed-effect ANOVA (see Results and Discussion). We suggest that instead of focusing on the binary classification of significant and nonsignificant results, more attention be drawn to effects with large effect sizes, even those with *p*-values larger than the conventional threshold of 0.05 (in analogous cases, 95% C.I.s that included zero in our results) [55–57].

All statistical analyses were performed in R version 3.4.4 [63] (R scripts available at https://osf.io/chabj/). Bootstrap resampling was performed using the boot function in the “boot” package (v. 1.3–20) [64]. The mixed-model ANOVA was performed with the aov function in the R base package.

#### Estimating effect sizes and confidence intervals

We first examined the fraction of seed germination and mortality for each plant species after inoculation by each fungal strain. To do so, we determined the effect size of these two responses, which was the difference in the mean fraction of germination/mortality between inoculated seeds and uninoculated controls. The mean fraction was averaged over five replicates in the same temperature regime. We then used 10,000 repetitions of non-parametric bootstrapping to determine the 95% C.I. of the effect size for each strain. In each repetition, we sampled with replacement the five replicates in each group of inoculated seeds and those in the control group, and calculated the resampled effect size. We assumed a two-tailed, equal-tailed distribution of each bootstrapped population of an effect size and calculated its 95% C.I. We used the same bootstrapping approach to obtain 95% C.I.s of mean fractions of seed germination and mortality in the control groups alone.

Next, we used the same general approach to obtain the effect sizes and C.I.s of the differences between the two plant species in their seed germination and mortality as a response to the same fungal strain. Any non-zero difference indicated by the 95% C.I. suggests host-specific seed germination or seed mortality, a pathway with the potential to influence plant coexistence (see Results and Discussion). The effect size was the difference between the two species in their effect size of seed germination/mortality after inoculation by the same fungal strain (see paragraph above). To acquire the 95% C. I. of each effect size, in each of the 10,000 repetitions, we sampled with replacement the five replicates in each group of inoculated seeds and those in the control group for both plant species, and calculated the resampled effect size. Responses of seeds of both plant species in the same Petri plate always were resampled together. Hence, our resampling algorithm mimicked the sampling method in the experiment, meeting an essential requirement of bootstrap [59]. Other assumptions in our bootstrapping method were the same as mentioned above.

Finally, we evaluated the degree to which each fungal strain could cause host-specific seed loss. Non-host-specific seed loss caused by fungi may undermine plant coexistence by increasing plant niche overlap related to apparent competition; or promote plant coexistence if this intensified apparent competition can be differentiated between plant species through environmental variation (see Results and Discussion) [21]. Host-specific seed loss may either promote or undermine plant coexistence, depending on whether the more dominant plant species is more or less limited by higher seed losses, and whether the density-dependent feedback among plant species could be differentiated by the host-specific pathogens (e.g., [3]). We examined the overall effects of each fungal strain on the loss of the seed bank for each plant species, defined by the combined fraction of seed germination and mortality in the summer temperature regime. Excluding physical displacement, germination and mortality are the only ways for a seed to leave the seed bank [7,28]. Seed bank loss due to winter germination is usually compensated by seed recruitment in the spring season. In contrast, summer germination of winter annuals is usually lethal–a loss to the seed bank that is further compounded by actual seed mortality. As seed mortality was generally not observed in the winter temperature regime in our experiment (see Results and Discussion), we considered losses to the *in vitro* seed bank as those occurring through germination and seed mortality in the summer temperature.

We defined the effect size of the seed loss of each plant species as the mean difference in this combined fraction between inoculated and control seeds in the summer temperature regime. We obtained the 95% C.I. of each effect size via bootstrap (above). We then further defined the effect size of host-specific seed loss as the difference between the two plant species in their effect size of seed loss to the same fungal strain. We used the same bootstrap method to obtain the 95% C.I. of the effect size of host specificity. Seed losses of both plant species in the same Petri plate always were resampled together.

#### Significance tests

We logit-transformed the fractions of seed germination, mortality, and loss. We then performed *t*-tests for every inoculated seeds-versus-control comparison. To accommodate a fraction of 0 and 1 in logit functions, we added the smallest non-zero fraction of germination, mortality, and seed loss (0.1 in all cases) to the numerator and the denominator of all odds ratios. Graphical examination of the transformed data showed this modification sufficiently avoided introducing outliers to the analysis [65].

Next, we used a mixed-model ANOVA to test for significant fungal strain × plant species interactions on (logit-transformed) fractions of seed germination, mortality, and loss. Each Petri plate was treated as a random effect. In the winter temperature regime, no biologically meaningful seed mortality was detected (see Results and Discussion) and analyses were restricted to seed germination.

## Results and discussion

**Fig 1:**
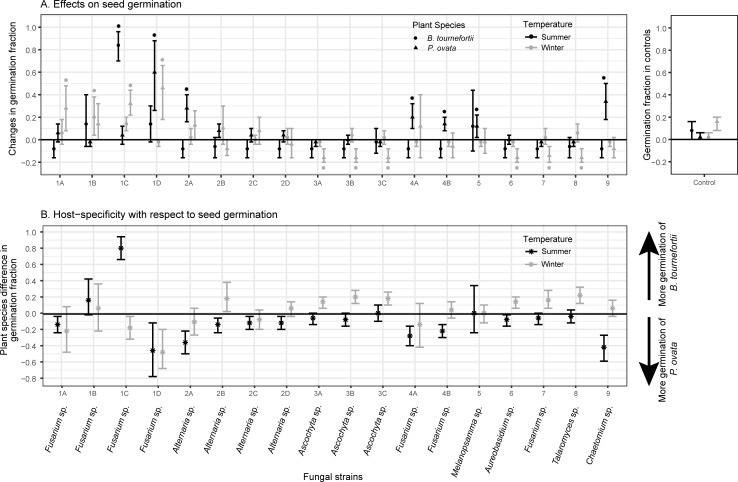
**(Panel A) Effects on the fractions of seed germination differed strongly between the fungal strains and between the summer (black markers) and the winter (grey markers) temperature regimes. (Panel B) Host-specific effects on seed germination were observed in both temperature regimes.** Fractions of germination of uninoculated seeds (controls) are shown as inserts on the right. In Panel A, effect sizes of germination were estimated as average changes in the fractions of germination between seeds inoculated by a fungal strain vs. controls. Asterisks indicate effects that were significant according to *t*-tests (*α* = 0.05; *p*-values of *t*-tests in S2 Table). In Panel B, effect sizes of host-specificity were estimated as the differences between the two plant species in their effect sizes of seed germination in response to the same fungal strain. In both panels, bars indicate 95% confidence intervals of effect sizes obtained by nonparametric bootstrapping. Taxonomic assignments of fungal strains to genus levels were provided by T-BAS [50].

**Fig 2:**
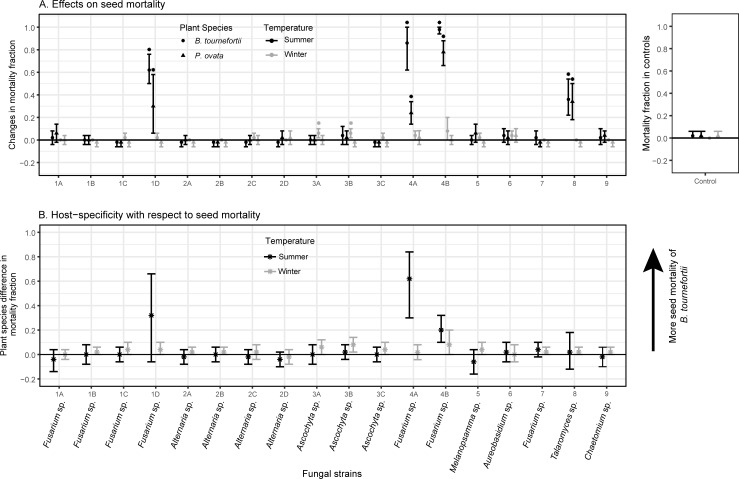
**(Panel A) Effects on the fractions of seed mortality differed strongly between the fungal strains and were more pronounced in the summer (black markers) than in the winter (grey markers) temperature regime. (Panel B) Host-specific effects on seed mortality were observed only in the summer temperature regime, and largely toward *B*. *tournefortii*.** Fractions of mortality of uninoculated seeds (controls) are shown as inserts on the right. In Panel A, effect sizes of seed mortality were estimated as average changes in the fractions of mortality between seeds inoculated by a fungal strain vs. controls. Asterisks indicate effects that were significant according to *t*-tests (*α* = 0.05; *p*-values of *t*-tests in S2 Table). In Panel B, effect sizes of host-specificity were estimated as the differences between the two plant species in their effect sizes of seed mortality in response to the same fungal strain. In both panels, bars indicate 95% confidence intervals of effect sizes obtained by nonparametric bootstrapping. Taxonomic assignments of fungal strains to genus levels were provided by T-BAS [50].

Representative fungi isolated from seeds of an invasive winter annual in the Sonoran Desert, *B*. *tournefortii*, had temperature-dependent and host-specific effects on the fraction of seed germination and mortality in that species and a co-occurring, native winter annual species (*P*. *ovata*) (Fig 1, Fig 2, Table 1, S2 Table).

### Effects under the winter temperature regime

Under the winter temperature regime, seed mortality due to the inoculation by fungi was minimal. Both 95% C.I.s and *t*-tests indicated that only *B*. *tournefortii* seeds exposed to strains 3A and 3B (*Ascochyta* sp.) had a non-zero increase in seed mortality by 6% (Fig 2A, grey markers). These small effects in increasing mortality were specific to *B*. *tournefortii* (Fig 2B, grey markers). Many fungal strains induced non-zero increase or reduction in seed germination (Fig 1A, grey markers). The fraction of seed germination reflected a fungal strain × plant species interaction (*F*_17,72_ = 4.9, *p* = 7.88×10^−7^), suggesting a substantial degree of host specificity that differed between fungal strains. Seven strains (i.e., 2B (*Alternaria* sp.), 3A, 3B, 3C (*Ascochyta* sp.), 6 (*Aureobasidium* sp.), 7 (*Fusarium* sp.), and 8 (*Talaromyces* sp.)) favored more germination of *B*. *tournefortii* than *P*. *ovata* (Fig 1B, C.I.s of grey markers), all through reducing germination of *P*. *ovata* seeds (Fig 1A). The effect sizes of these inter-host differences generally did not exceed a germination fraction of 20% (Fig 1B, grey markers). Two strains (1C and 1D, *Fusarium* sp.) favored more germination of *P*. *ovata* than *B*. *tournefortii* (Fig 1B, C.I.s of grey markers) largely through a stronger increase in *P*. *ovata* germination (Fig 1A). In particular, strain 1D induced 48% more germination of *P*. *ovata* than *B*. *tournefortii* seeds (Fig 1B, grey marker).

While some of the fungi in our inoculation experiment showed effects of reducing winter germination of the two plant species, our experiment might not fully demonstrate these effects. The inoculation experiment was performed in April, when seeds of these species typically start to become dormant (or conditionally dormant; see [32]). The seeds were not exposed to the high ambient temperature in the summer prior to the experiment, and thus might have been more likely to retain dormancy. In the winter temperature regime, the mean germination fraction of *B*. *tournefortii* and *P*. *ovata* in the control groups was only 2% and 16%, respectively (Fig 1A, right insert), such that detecting effects of fungi in reducing germination would be difficult, and a comparison of such effects between the two plant species could be problematic.

Our sterilization procedure did not eliminate all fungi from seed surfaces prior to the initiation of the inoculation experiment. One unidentified strain was observed in the controls and to seeds inoculated by some of the focal fungal isolates. This fungus did not affect the fraction of seed germination or mortality in control seeds, which remained indistinguishable from zero except for the winter germination of *P*. *ovata*, which increased (Fig 1A, right insert). This increase did not appear to be attributed to this unidentified fungus, because a reduction in winter germination was observed in the only other group of *P*. *ovata* seeds (those inoculated by strain 7; see Fig 1A) in which this fungus was observed. The identity and potential importance of this fungus will be evaluated in future work.

Despite these caveats, our result showed that strains 1C and 1D increased winter germination of *P*. *ovata* more than that of *B*. *tournefortii* (Fig 1B, grey markers), inducing plant species-specific germination fractions. Under natural conditions, germination of desert winter annual plants is sensitive to cool-season temperatures, soil moisture levels, light availability, and other abiotic factors [31,33,34]. Our findings showed that, under laboratory conditions, seed germination fraction of desert winter annuals could be affected by soil-borne fungi, and these effects differed between plant species. Coexistence of diverse desert winter annuals often is attributed to temporal or spatial niche differentiation, which operates through plant species-specific germination responses (e.g., germination fractions and speeds) to a variable environment [28,30,31]. Our results suggest that the presence and identity of fungi may act as additional environmental factors that can yield distinctive germination responses, creating a possible pathway of niche differentiation that may promote plant species coexistence. This finding is consistent with those from more mesic systems in which soil-borne microbes cause host-specific physiological responses in plants with demographic and community-level effects [11,17,18,66].

In southwestern Arizona, *P*. *ovata* seeds had lower fractions of germination than those of *B*. *tournefortii* on a sand flat, but higher fractions on a dune, and these differences were more pronounced when the amount of first winter rainfall increased [33], which is a more mesic condition that may favor fungi growth. It would be meaningful to examine whether dominance of certain fungal strain on the dune, with an effect analogues to strains 1C or 1D, could contribute to this environmental differentiation in the germination responses between the two plant species.

### Effects under the summer temperature regime

Under the summer temperature regime, many fungal strains increased the fractions of seed germination and mortality of either plant species (Figs 1A and 2A, black markers). Fractions of seed germination and mortality both reflected a fungal strain × plant species interaction (respectively: *F*_17,72_ = 13.09, *p* = 9.48×10^−15^; *F*_17,72_ = 5.52, *p* = 1.12×10^−7^), suggesting these effects on the two determinants of seed bank losses differed between fungal strains, and some of these effects were host-specific (Figs 1B and 2B, C.I.s of black markers).

In particular, strain 1C (*Fusarium* sp.) caused 80% more summer germination of *B*. *tournefortii* than *P*. *ovata* seeds (Fig 1B, black marker). On the other hand, nine strains (1A, 1D (*Fusarium* sp.), 2A, 2B, 2C, 2D (*Alternaria* sp.), 4A, 4B (*Fusarium* sp.), and 9 (*Chaetomium* sp.)) caused more summer germination of *P*. *ovata* than *B*. *tournefortii* seeds (Fig 1B, C.I.s of black markers). The host-specific effects of these nine strains were generally small or moderate, with strain 1D causing the largest effect of inducing 46% more germination of *P*. *ovata* vs. *B*. *tournefortii* seeds (Fig 1B, black marker). Four fungal strains (1D, 4A, 4B *(*all *Fusarium* sp.), and 8 (*Talaromyces* sp.) caused moderate to high seed mortality on both plant species in the summer temperature regime (Fig 2A, black markers). Among them, strains 4A and 4B caused, respectively, 62% and 20% more mortality of *B*. *tournefortii* seeds than *P*. *ovata* seeds (Fig 2B, black markers). Strain 1D caused 32% more mortality of *B*. *tournefortii* than *P*. *ovata* seeds, but the 95% C.I. of this host-specific effect included zero (Fig 2B, black marker) likely due to both small sample sizes and a large variance within the five replicates of *P*. *ovata* seeds (Fig 2A).

The observed host-specific effects on seed mortality under summer conditions agreed with findings in a previous study of desert plants in the Great Basin ([10],see also [17] for a case in a tropical environment). Fungi also induced germination in the summer temperature regime, which represents a season when these plants are unlikely to survive after germination. The question remains whether summer germination could represent a mechanism for escape from fungal pathogens [10], and if so, whether this putative escape mechanism can be triggered in an intermediate temperature regime that is less hostile to germinated plants than the summer conditions tested here.

**Fig 3:**
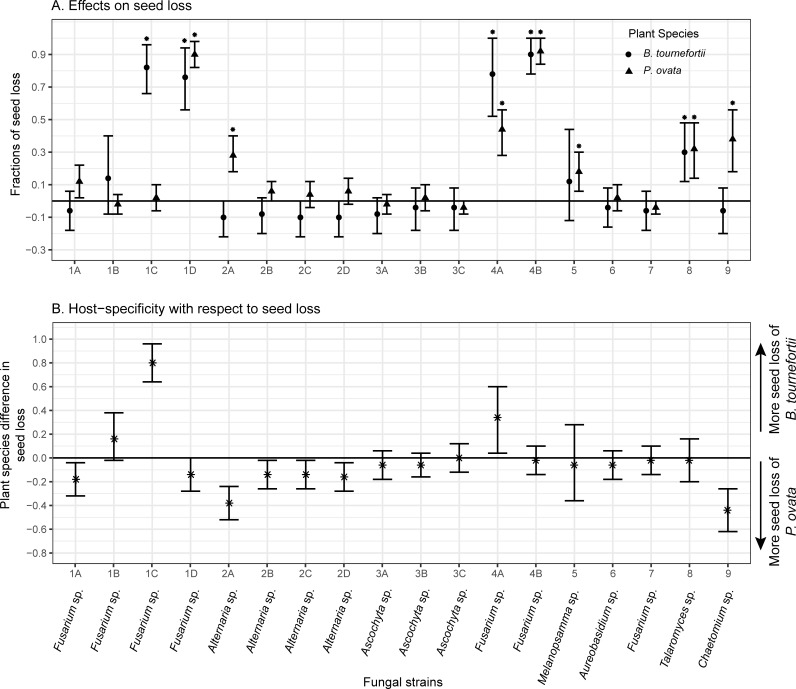
**Some fungi caused losses of the *in vitro* seed banks (Panel A), and some of these effects were host-specific (Panel B).** In Panel A, effect sizes of seed loss were estimated as average changes in the combined fractions of seed mortality and germination in the summer temperature regime between seeds inoculated by a fungal strain vs. controls. Asterisks indicate effects that were significant according to *t*-tests (*α* = 0.05; *p*-values of *t*-tests in S2 Table). In Panel B, effect sizes of host-specificity were estimated as the differences between the two plant species in their effect sizes of seed loss in response to the same fungal strain. In both panels, bars indicate 95% confidence intervals obtained via nonparametric bootstrapping. Taxonomic assignments of fungal strains to genus levels were provided by T-BAS [50].

The combined effect of increasing seed mortality and germination under the summer temperature regime (when seedling survival is unlikely) is a loss to seed bank that can reduce population growth of annual plants [31]. Our *in vitro* trials demonstrated that 9 of the 18 strains (1A, 1C (*Fusarium* sp.), 2A (*Alternaria* sp.), 4A, 4B (Fusarium sp.), 5 (*Melanopsamma* sp.), 8 (*Talaromyces* sp.), and 9 (*Chaetomium* sp.)) caused seed losses of either or both plant species (C.I.s in Fig 3A). Fractions of seed loss reflected a fungal strain × plant species interaction (*F*_17,72_ = 13.06, *p* = 9.97×10^−16^), suggesting host-specific effects on seed losses, the degree to which differed between the fungal strains. Among these strains, strains 1C and 4A caused more seed losses of *B*. *tournefortii*, whereas strains 1A, 2A, 2B, 2C, 2D, and 9 reduced *P*. *ovata* seeds more strongly (C.I.s in Fig 3B). Although more fungal strains caused higher seed losses of *P*. *ovata*, their effects were generally small or moderate. The highest degree of host-specificity to *P*. *ovata* was induced by strain 9, with an effect size of an additional fraction of 44% seeds lost by *P*. *ovata* vs. *B*. *tournefortii* (Fig 3B). In comparison, the two strains specific to *B*. *tournefortii* increased the plant’s seed loss by a fraction of 80% (strain 1C) and 34% (strain 4A) of seeds relative to *P*. *ovata*. Overall, strain 1C (*Fusarium* sp.) appeared to be the most specific seed pathogen to *B*. *tournefortii*, whereas strain 9 (*Chaetomium* sp.) appeared to be the most specific to *P*. *ovata* (Fig 3B). Both strains caused seed loss of their specific hosts through inducing summer germination (Fig 1A, black markers).

### Implications for plant species coexistence

Overall, our results show that fungi that infect seeds of an invasive plant species can change fractions of seed germination and increase seed losses of both that invasive species and a co-occurring, native plant. The observed host-specific and non-host-specific effects of these fungi suggest potential impacts on demographic processes relevant to the coexistence of winter annual plants in warm deserts with bimodal annual rainfall. The capacity of fungi associated with seeds of an invasive species to increase or decrease the fraction of winter germination and reduce the *in vitro* seed bank of a native plant points to cryptic but potentially important impacts of invasive plants on species-rich communities of desert winter annuals.

According to the coexistence theory, the invasion of an introduced species can be attributed to either a higher average fitness of this species or a stabilizing mechanism (sometimes regarded as a niche opportunity) that enables a positive invasion rate of that species [43,44]. Furthermore, dominance of an invasive species is attributed to its higher average fitness relative to other species [43,44]. Stabilizing mechanisms are required to overcome that elevated average-fitness difference in order for other species to coexist with the species in dominance.

Assuming *B*. *tournefortii* is the dominant species in an annual plant community, a scenario common at the advanced stages of plant invasions, a dominance of fungal strains specific to seed losses of *B*. *tournefortii* (e.g., strain 1C) may reduce the average-fitness advantage of *B*. *tournefortii* over native species such as *P*. *ovata*, making it more likely for the invasive and native plant species to coexist. On the other hand, if strains specific to the loss of *P*. *ovata* seeds dominate (e.g., strain 9), *B*. *tournefortii* may gain further average-fitness advantage over *P*. *ovata*. Coexistence hence would become less likely [3,19]. Further, if seeds of both plant species are affected by host-specific fungal pathogens, the effect may be consistent with natural enemy partitioning, promoting coexistence (sensu [9,13,15–17]).

Seeds of *B*. *tournefortii* in southwestern Arizona had consistently higher mortality than those of *P*. *ovata* and other common winter annual species, over three years and three different types of habitat covering approximately 0.5 km^2^ [33]. The population density of *B*. *tournefortii* declined dramatically over the three years on that landscape scale, while those of the other winter annual species remained relatively stable. This sharper decline of *B*. *tournefortii* population was partially due to the higher seed mortality of this species [33]. We speculate that the aforementioned demographic dynamics may be partially explained by a dominance of fungi that cause higher seed loss to *B*. *tournefortii* than to other annual plants, analogous to strain 1C and 4A (*Fusaria* sp.; Fig 3B) isolated in our study. Such dominance of host-specific seed pathogens would have reduced the average fitness of *B*. *tournefortii*, promoting the coexistence in that annual plant community.

In addition to host-specific seed losses, some fungi in our study caused substantial seed losses in both species (e.g., strains 1D, 4B, and 8; see Fig 3). This non-host-specific effect, under natural conditions, may intensify apparent competition, which, depending on the ecological context, can have polarizing influences on plant coexistence [7,20,21]. Contrary to conventional wisdom, apparent competition due to generalist pathogens may promote plant coexistence if this intensification of density dependent feedback via shared natural enemies further concentrates intraspecific density dependence relative to interspecific density dependence [21]. This can happen when plant species differ in their physiological responses to a variable environment (e.g., plant species-specific germination responses), and pathogen-induced apparent competition closely tracks these response differences to strengthen *the storage effect* [7,21,67]. This scenario happens when, for example, a pathogen attacks plants immediately after seed germination (e.g., seedling pathogens) [21]. Seedling pathogens are common in nature [68], though it is beyond the scope of this study to test whether any of the isolated strains could attack annual plant seedlings.

Apparent competition due to generalist pathogens also can weaken annual plant coexistence. First, it may increase niche overlap via natural enemies and weaken niche differentiation via resource competition [7,20,69], especially when the pathogen-induced density dependence cannot track closely the interspecific differences in plant physiological responses to a variable environment [7,69]. This can happen if fungal pathogens kill annual plant seeds before seed germination, as demonstrated in our laboratory experiment. Second, seed losses due to pathogens weaken seed banks of annual plants. Seed banks prevent population crashes over unfavorable periods, and thus contribute to buffered population growth, an essential component of the temporal storage effect [30,69]. Reducing seed banks weakens the temporal storage effect, undermining coexistence of desert annual plants in communities that are primarily stabilized by this coexistence mechanism [21].

Two fungal strains (1D, *Fusarium* sp., and 8, *Talaromyces* sp.) in our study could be seed pathogens (increasing summer seed mortality; Fig 2A, black markers) while also inducing plant species-specific winter germination (Fig 1B, grey markers). This finding challenges the conventional assumption that the germination response (e.g., germination fraction) of desert winter annual seeds is primarily influenced by abiotic factors independent of seed density (e.g. temperature and soil moisture level). This assumption of density-independent germination is used in models explaining coexistence of annual plants via the storage effect [28,30]. Our finding implies that as seed pathogens, the densities of fungi like strains 1D and 8 may follow those of their hosts. Their host-specific effects on winter germination raise the possibility that germination fraction of a plant species may depend on the density of competing plant species in the field. In a hypothetical scenario when *B*. *tournefortii* is more dominant than the native *P*. *ovata*, a high seed density of *B*. *tournefortii* could lead to a high density of a seed pathogen, which could either increase (e.g., strain 1D) or reduce (e.g., strain 8) germination of the less dominant *P*. *ovata*, either intensifying or reducing the competition between *P*. *ovata* and *B*. *tournefortii*, hence weakening or strengthening their coexistence. To evaluate whether the assumption of density-independent germination responses can largely stay unchallenged, field studies are needed to assess the prevalence of fungi that are seed pathogens but also affect germination of co-occurring plant species.

### Future work

In this study we took a reductive approach and showed that when studied in simplified artificial conditions and in isolation, fungal strains differed in the degree to which they were host plant specific, and in their impact on seed germination and mortality. Our use of a rich medium such as MEA might alter fungal growth and traits, perhaps shifting fungi to a more pathogenic lifestyle [70]. Nevertheless, our finding that certain fungi caused higher loss in seeds of *B*. *tournefortii* than *P*. *ovata* was consistent with field evidence of higher seed mortality of *B*. *tournefortii* [33]. To extend the scope of the current study, one important next step is to include inoculation trials in soil under natural field conditions.

Winter annual plants in the warm Sonoran and Chihuahuan Deserts have long been used as a model system to test species coexistence theory, especially with regard to stabilizing mechanisms such as the storage effect [28,30] and frequency dependent predation [71]. Yet, we are unaware of any study that has examined the role of fungi in influencing species coexistence in this model system. Understanding the overall effects of fungi on species coexistence in this model system will require weaving theoretical investigation tightly with reductive controlled experiments and inductive observational studies. As the impacts on seed germination and mortality vary by individual fungal strains, their net impact on these seed demographic rates will depend on the composition of a fungal community. To make the investigation more challenging, not only does fungal community composition varies in time and space [48], impacts of individual fungal strains on plant demography will interact with a variable environment to change over time and space as well [70]. Furthermore, the influence of fungi on plant coexistence through host-specific or non-host-specific effects largely depend on the ecological context, such as whether the more dominant plant species experiences more host-specific seed losses [19], or whether pathogen-induced apparent competition can closely track interspecific differences in plant physiological responses to a variable environment [7,21].

This context dependency underscores the use of theoretical models to simulate different ecological scenarios and to evaluate the net influence of fungi on plant coexistence in each context (e.g., [19,21]). Controlled experiments situated in a comparable ecological context can ensue to examine whether species coexistence outcomes would support theoretical predictions [72]. Yet, such experiments, even given the most state-of-art technology and abundant human resources, would hardly reach the complexity of a natural environment. Hence, large-scale and long-term observational studies in a non or minimally manipulated environment, where the composition of fungi and plant demography are systematically surveyed and analyzed, would be the foundation for further theoretical and reductive empirical inquiries [73]. Overall, studying the effects of soil-borne fungi and other microbes on plant species coexistence in arid environments will help to build broader comparisons across temperate and tropical systems and thus illuminate the influence of fungi on plant diversity on a global scale.

## Supporting information

S1 Table Fungal strains and their attributes. T-BAS [50] was used to define OTUs (based on 95% sequence similarity), groups based on 99% similarity, strains (genotypes) based on 100% sequence similarity, and taxonomic placement. Names of the first eighteen 95% OTUs match the strain names given in Table 1. The sources of the fungi are viable (V), degraded (D), or both (V&D) seeds of *B*. *tournefortii*. GenBank accession numbers are given for all isolates.
(CSV)

S2 Table Effects of 18 fungal strains on seed germination and mortality of *B*. *tournefortii* and *P*. *ovata* in the summer and winter temperature regimes, as well as the effects on seed loss (germination + mortality in the summer temperature) of the two plant species. An effect size was calculated as the difference in the mean fraction of germination, mortality, or loss between seeds inoculated with a fungal strain and those uninoculated as a control group. Columns 5–8 present statistics of pairwise *t*-tests: the effect size as the fractions were logit-transformed (see Methods), the standard error of the effect size, the *t*-statistic, and the *p*-value. Column 9–12 present the untransformed effect size, the lower and upper bound of its 95% confidence interval (C.I.) estimated by nonparametric bootstrapping (see Methods), and the bias of the bootstrapped effect size.
(CSV)
